# Primary hepatocellular adenoma due to biallelic *HNF1A* mutations and its co-occurrence with MODY 3: case-report and review of the literature

**DOI:** 10.1007/s12020-019-02138-x

**Published:** 2019-11-21

**Authors:** Junling Fu, Tong Wang, Xiao Zhai, Xinhua Xiao

**Affiliations:** Department of Endocrinology, NHC Key Laboratory of Endocrinology, Peking Union Medical College Hospital, Chinese Academy of Medical Sciences and Peking Union Medical College, Beijing, 100730 China

**Keywords:** *HNF1A*, MODY 3, Hepatocellular adenoma

## Abstract

**Purpose:**

Maturity-onset diabetes of the young type 3 (MODY 3) is a consequence of heterozygous germline mutations in *HNF1A*, and a subtype of hepatocellular adenoma (HCA) is caused by biallelic somatic *HNF1A* mutations; rare HCA may be related to MODY 3. This study aimed to investigate the cosegregation of *HNF1A* mutations with diabetes and HCA in two families.

**Methods:**

Two patients suffering from HCA and diabetes were screened for *HNF1A* germline and somatic mutations using direct sequence analysis and methylation-specific multiplex-ligation-dependent probe amplification (MS-MLPA) assay. Further, we screened eight relatives in the two independent families for diabetes, HCA and *HNF1A* variants. Additionally, we reviewed the literature concerning the phenotypes of MODY 3 and HCA at the background of *HNF1A* mutations.

**Results:**

Here we reported two families (a total of six relatives) with two missense germline mutations of *HNF1A* identified initially using direct sequence analysis (c.686G>A in family A and c.526 + 1G>A in family B). Somatic deletion of the second allele of *HNF1A* was found in liver tumor tissues in both probands who were diagnosed with HCA. There are a total of ten cases of both MODY 3 and HCA phenotypes reported in the literature to date; incomplete penetrance for HCA was observed, and all the patients with HCA developed diabetes. The onset of diabetes and HCA was highly variable, the treatment of diabetes varied from diet to insulin, and the clinical expression of HCA ranged from silent to hemorrhage. Further, the severity of diabetes mellitus was not related to the occurrence of HCA.

**Conclusions:**

This study describes the association of HCA and MODY 3 at the background of *HNF1A* mutations and highlights the importance of screening for HCA in MODY 3 families to avoid the possibility of severe complications. Further, the current study indicated that there may be a special mutational spectrum of *HNF1A* correlated with HCA in MODY 3 families.

## Introduction

Maturity-onset diabetes of the young (MODY) is an autosomal dominantly inherited type of diabetes that results from heterozygous mutations in various transcription factors acting in the development and function of pancreatic beta cells [1]. MODY 3 is caused by inactivating germline heterozygous mutations in the hepatocyte nuclear factor 1a (*HNF1A*) gene, which accounts for nearly 2–5% of insulin independent diabetes and 30–65% of MODY and is characterized by positive glycosuria, microvascular complications tendency, and sulfonylurea priority [2]. *HNF1A* is mainly expressed in pancreatic beta cells, the intestine, and the liver [1], and plays an important role in the cellular function to regulate glycolipid metabolism. Mutations in *HNF1A* lead to decreased insulin secretion, and progressive damage of beta cells and thus cause the onset of diabetes. Hepatocellular adenoma (HCA), which is known as a liver tumor characterized as being rare and benign, is widely believed to be related to oral contraception [3], and usually manifests as a single tumor, however, when several adenomas are discovered in one patient, this HCA is termed liver adenomatosis [4]. Regardless of solitary or multiple, HCAs can be complicated by ache, hemorrhage, and increased risk of hepatocellular carcinoma. HCAs can be classified into molecular subtypes; ~30% of HCAs are associated with the inactivation of *HNF1A*, and 90% of the cases are somatic mutations [5]. Thus, we hypothesized the existence of a relationship between *HNF1A* mutations and phenotypes of both MODY 3 and HCA, sometimes with the two pathologies appearing in the same individuals [6–9]. To date, there have been four reports, and a total of ten cases showing the connections between the two phenotypes in subjects with *HNF1A* mutations.

We herein report the cosegregation phenotypes of HCA and diabetes in two unrelated Chinese MODY 3 families with germline and somatic *HNF1A* mutations, and reviewed the literature concerning phenotypes of MODY 3 and HCA.

## Subjects and methods

### Subjects

The probands from two families exhibited early-onset noninsulin-dependent diabetes as well as HCA. HCA was discovered by noninvasive radiological imaging, and confirmed by histological evaluation (Fig. 4), which was performed from surgical specimens. The diagnosis of diabetes was based on the diagnostic criteria of the American Diabetes Association [10]. Screening for HCA was based on liver ultrasonography and/or computed tomography (CT).

### DNA extraction

Peripheral blood samples (4 ml) were obtained from the two MODY probands and the relatives available from the two families. Genomic DNA was extracted from whole blood using a QIAamp DNA Mini Kit (Qiagen China Co., Ltd., Shanghai, China) according to the manufacturer’s recommendations [11].

Liver tumor tissue samples collected from two probands with proven HCA by surgical biopsy were frozen immediately in liquid nitrogen and stored at −80 °C. Tumor DNA was extracted using a salting-out procedure. Informed consent was obtained from all individual participants and/or parents/guardians included in the study.

### Germline *HNF1A* mutation analysis

The ten exons, the exon–intron boundaries, and the promoter region of *HNF1A* were screened for mutations in the two probands by direct sequencing of PCR products as previously described [11]. The NCBI BLAST database was used to identify variants by aligning with reference sequences NM_000545.6 (*HNF1A*). The direct diagnosis of the mutation identified in the probands were then offered to relatives independently of their clinical status.

### Somatic *HNF1A* mutation analysis

The direct sequencing of PCR products and the methylation-specific multiplex-ligation-dependent probe amplification (MS-MLPA) assay were performed in tumor DNA. MS-MLPA was performed with SALSA MLPA Probemix P241-E1 MODY Mix 1 (MRC-Holland, Amsterdam, The Netherlands) in Beijing Novocardio Biotechnology Co., Ltd. The P241-E1 MODY Mix 1 Probemix contains 52 MLPA probes with amplification products between 130 and 500 nt. It contains probes for the *HNF4A, GCK, HNF1A*, and *HNF1B* genes and is therefore specific for MODY 1, 2, 3, and 5. For the *HNF4A* gene, 12 probes are included, furthermore 11 for the *GCK* gene, 11 for the *HNF1A* gene, and 10 for the *HNF1B* gene are included. In addition, eight reference probes are included in this probe mix. The identity of the genes detected by the reference probes is available online (www.mlpa.com). The validation experiment should result in a standard deviation <0.10 for all probes over the experiment (0.08 for proband 1, 0.05 for proband 2). The threshold for chromosomal abnormalities was established as follows: the lower limit was 0.7 for deletion and the upper limit was 1.33 for duplication. Abnormal values were plotted outside the threshold line. Probes for *HNF1A* are listed in Supplementary Table 1.

### Literature review

The literature search occurred in March 2019. We systematically identified all potentially relevant articles from the following three electronic databases: MEDLINE, PubMed and Web of Science. Search terms about diabetes such as “maturity-onset diabetes of the young (MODY)” and “*HNF1A-MODY*”, and liver neoplasms, such as “liver tumor”, “hepatocellular adenoma” and “liver neoplasms”, were used in various combinations and permutations across the databases. Language restriction (English) was applied. There were a total of four papers that included ten patients that met the requirements [6–9] (Table 2).

## Results

### MODY expression

**Fig. 1:**
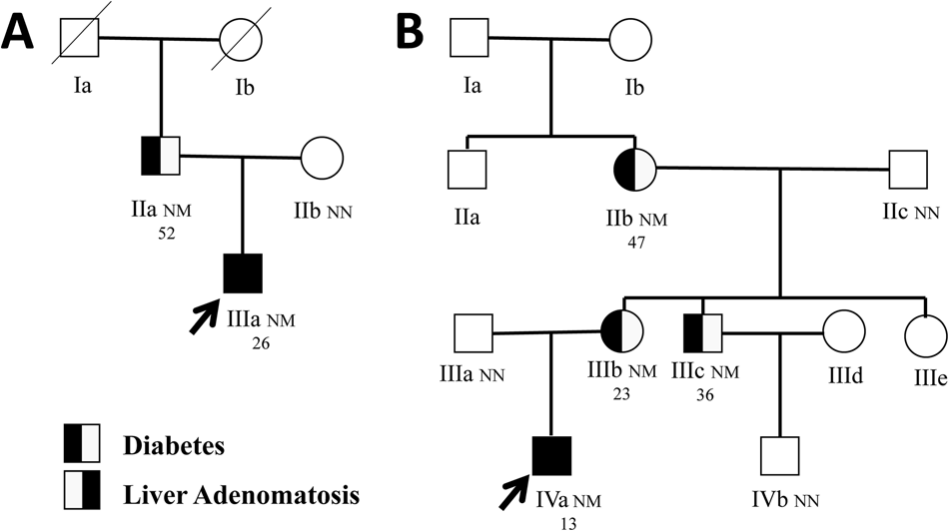
Pedigree of families of proband 1 (**a**) and proband 2 (**b**). Squares represent male family members, while circles represent female family members. The black symbol on the left represents individuals with diabetes, the black symbol on the right represents individuals with hepatocellular adenoma, and the blank symbols represent normal individuals. Arrows indicate probands in the families (**a**, IIIa; **b**, IVa). Variant carrier status is presented as N: Normal allele and M: Mutation. The sequence data displayed heterozygous mutations in *HNF1A* (c.686G>A, p.R229Q in proband 1; c.526 + 1G>A in proband 2). The second line under the symbols corresponds to the onset age of diabetes

**Fig. 2:**
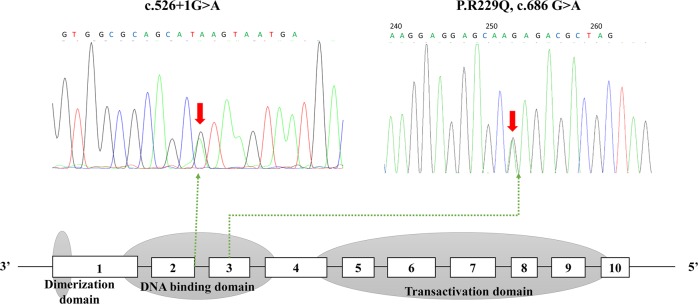
The sequencing chromatogram and position of the mutation in *HNF1A* gene. Arrows indicate the changed nucleotide

The familial history of diabetes and the age at diagnosis of diabetes in both families suggested that the two probands may be diagnosed as MODY (Fig. 1; Table 1). The screening of *HNF1A* was therefore initiated in the two probands (Fig. 2). A heterozygous mutation (c. 686G>A, p. Arg229Gln) in *HNF1A* was found in proband 1. The mutation caused a change in the protein from Arg to Gln at p. Arg229, which is located in exon 3 of *HNF1A*. The mutation was subsequently searched for in other available family members in family 1, and was also found in the father. A heterozygous mutation (c.526 + 1G>A) in *HNF1A* was found in proband 2, and in other three relatives in family 2, and the mutation cosegregated with the clinical phenotypes of MODY 3 within the pedigree. The mutation is in the first nucleotide of intron 2 in *HNF1A*, and this variant is a splicing mutation that may affect protein function and cause diabetes. Neither of the two mutations were found in the ExAC database [12].

**Table 1:** Laboratory investigation

Laboratory (serum)	Proband 1	Proband 2	Normal range
Sex	M	M	/
Age (year)	32	19	/
Birthweight (kg)	3	3.7	/
Height (cm)	175	186	/
Weight (kg)	68	70	/
Body mass index (kg/m^2^)	22.2	20.2	/
Systolic blood pressure (mmHg)	120	110	/
Diastolic blood pressure (mmHg)	80	75	/
Fasting glucose (mmol/L)	7.5	4.9	3.9–6.1
2 h-postprandial glucose (mmol/L)	9.1	11.3	/
Fasting C-peptide (ng/ml)	0.83	0.72	0.9–4.2
2 h-postprandial C-peptide (ng/ml)	4.3	3.75	/
Glycated albumin (%)	17.3	13.4	10.8–17.1
Glycated hemoglobin (%)	5.8	5.7	4.5–6.3
Current therapy	Sulfonylurea	Diet	/
Inheritance	Father	Mother	/
Total cholesterol (mmol/L)	4.04	3.7	2.85–5.7
Triglyceride (mmol/L)	0.8	0.54	0.45–1.7
HDL-C (mmol/L)	1.14	1.14	0.93–1.81
LDL-C (mmol/L)	2.28	2.19	<3.37
Hs-CRP (mg/L)	0.16	0.06	0–3.0
Alanine transaminase (U/L)	30	19	9–50
Aspartate aminotransferase (U/L)	27	19	15–40
Total bilirubin (umol/L)	18	17.2	5.1–22.2
Direct bilirubin (umol/L)	6.8	7.4	0–6.8
GGT (U/L)	11	17	10–60
Albumin (g/L)	47	50	35–52
Creatinine (umol/L)	84	81	59–104
Uric acid (umol/L)	192	349	210–416
AFP (ng/ml)	1.4	1.9	0–20
CEA (ng/ml)	0.76	0.37	0–5
CA199 (U/ml)	4.8	6.3	0–34
Routine urine			
GLU	NEG	NEG	NEG
PRO	NEG	NEG	NEG
KET	NEG	NEG	NEG
ACR (mg/g Cr)	3	4	0–30

*HDL-C* high-density lipoprotein cholesterol, *LDL-C* low-density lipoprotein cholesterol, *HOMA-IR* homeostasis model assessment of insulin resistance, *hsCRP* high sensitivity C reactive protein, *AFP* alpha-fetoprotein, *CEA* carcinoembryonic antigen

The clinical manifestations of diabetes in the two families were extraordinarily variable, the age of onset ranged from 13 to 52, and the treatment varied from diet to sulfonylurea. Further, they had BMI ranges from 19.2 to 24.3 kg/m^2^. Laboratory testing was only notable for elevated fasting glucose (7.5 mmol/L), 2 h-postprandial glucose (9.1 mmol/L) and glycated albumin (17.3%) in proband 1 and for elevated 2 h-postprandial glucose (11.3 mmol/L) in proband 2.

### Hepatocellular adenoma (HCA)

**Fig. 3:**
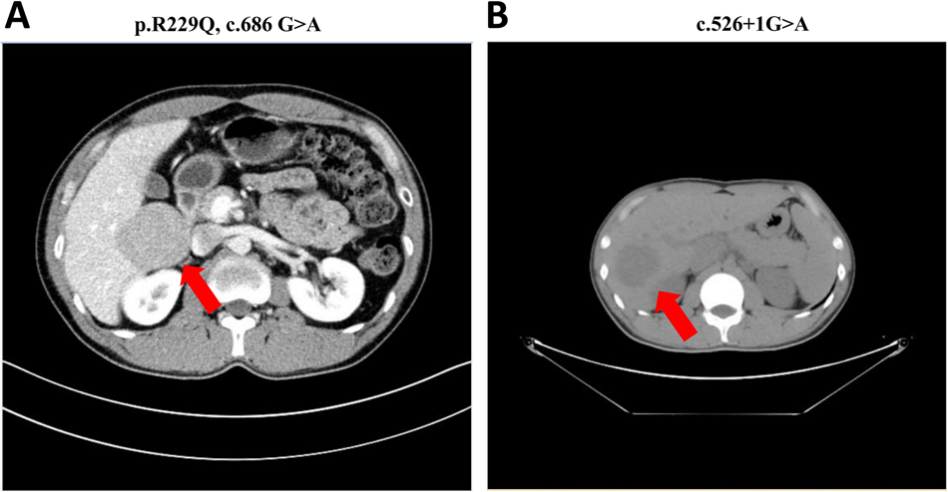
Computed tomography (CT) scans of the two probands. CT scans taken on the preoperative day (**a**: proband 1; **b**: proband 2). The size of the tumors of proband 1 was 60 mm × 57 mm × 48 mm (52 HU), and that of proband 2 was 47 mm × 42 mm (48 HU) (arrowhead)

HCA was detected in the two probands but was not in other relatives diagnosed with MODY 3. In both probands, patients did not have abdominal symptoms or liver enzyme disturbances, and therefore HCA was diagnosed through ultrasonography screening. The tumor markers (CA199, CEA and AFP) were normal in both probands. Computed tomography (CT) scans were performed in the two probands (Fig. 3). In proband 1, a round soft tissue density shadow with a clear boundary (60 mm × 57 mm × 48 mm) was identified, located next to the posterior segment of the right hepatic lobe and the inferior vena cava and protruded outside of the liver (the CT value was ~52 HU). In proband 2, an irregular low-density lesion (47 mm × 42 mm) in the right lobe of the liver (CT value was ~48 HU) was found.

**Fig. 4:**
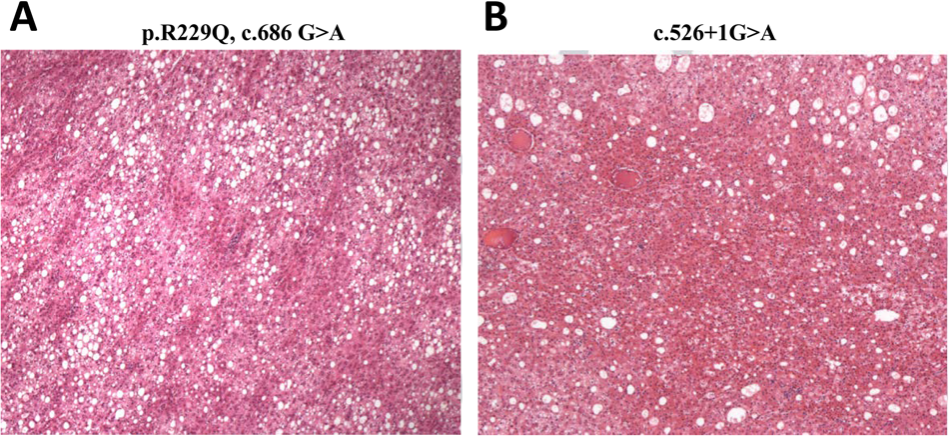
Histological characteristics of the hepatocellular adenoma. **a** proband 1: CD34 (part+), CK19 (−), CK7 (scattered+), Ki-67 (index 5%), β-catenin (membrane+), AFP (−), GPC-3 (−), and reticulum fiber (+). **b** proband 2: GPC-3 (−), CK7 (−), CEA (−), IMP3 (−), Ki-67 (index 2%), CD34 (vessel+), CAM5.2 (+), AFP (−), Hepatocyte (+), β-catenin (membrane+), and CK19 (−)

The two probands underwent hepatectomy and recovered uneventfully. HCA was confirmed by histology in both patients. Immunostains for AFP, glypican-3, and CK19 were both negative (Fig. 4). One-year follow-up was available in the two patients (Table 2). In proband 2, progressive growth of hepatic nodules was observed after a 1-year period of follow-up while no progression was observed in proband 1.

**Table 2:** Clinical characteristics of patients presenting with hepatocellular adenoma and diabetes mellitus and carrying the *HNF1A* mutations

	Diabetes								Hepatocellular adenoma			
	Sex	BMI (kg/m^2^)	Age at onset (y)	Current treatment	HbA1c (%)	Age at diagnosis (y)	Contraceptive use (y)	Clinical manifestations at diagnosis	Liver feature imaging at diagnosis	Duration of follow-up (y)	Evolution	Treatment
**Family 1**^c. 686G>A, p. Arg229Gln^												
**IIa**	M^no detection^	24.3	52	Sulfonylurea	7.6	/	/	/	/	/	/	/
**IIIa (Proband 1)**	M^deletion^	22.2	26	Sulfonylurea	5.8	31	No	None, US discovery	Solitary nodule 6 cm	1.0	Stable	Liver resection
**Family**^c.526+1G>A^												
**IIb**	F^no detection^	20.2	47	Sulfonylurea	/	/	/	/	/	/	/	/
**IIIb**	F^no detection^	19.2	23	Diet	6.2	/	/	/	/	/	/	/
**IIIc**	M^no detection^	23.1	36	Sulfonylurea		/	/	/	/	/	/	/
**IVa (Proband 2)**	M^deletion^	20.2	13	Diet	5.7	17	No	None, US discovery	Multiple nodules including a 5 cm adenoma	1.5	Progressive growth of nodules (3 cm)	Liver resection
[6] ^P291fs (872_873insC)^	F^no detection^		32	Insulin	/	17	No	Hepatomegaly	Multiple nodules including a 19 cm adenoma	22	Progressive growth of nodules (1–7 cm); intrahepatic bleeding	Right lobeotomy, then liver transplantation
	F^no detection^		24	Insulin	/	51	No	None, US discovery	Multiple nodules (0.5–2.5 cm)	7	Stable	None
	F^no detection^		15	Metformin	/	16	1.0	Ip bleeding of a 15 cm nodule	Massive liver adenomatosis (0.5–15 cm)	/	Death (16 y)	
	M^F277_H279del^		15	Insulin	/	15	/	None, US discovery	Multiple nodules (1–4 cm)	5	Stable	Liver resection
	F^deletion^		15	Nutritional advice	/	14	No	Hepatomegaly	Multiple nodules including a 12-cm adenoma	3	Stable	Liver resection
	F^no detection^		25	Nutritional advice (GD on insulin)	/	29	No	None, US discovery	Multiple nodules (0.3–1 cm)	<1.0	Stable	None
[7]^c.1340 C>T (p.P447L)^	F^no detection^		17	Insulin	/	55	10	None, US discovery	Multiple nodules (7–16 cm)	1.0	Stable	Liver resection
[8] ^ex2–3del^	M^no detection^		10	Sulponylurea	/	31	/	Rupture of a 10.5 cm liver mass	Multiple nodules (≤10.5 cm)	/	Stable	Left hepatectomy
	F^no detection^		7	/	/	56	/	None, US discovery	Solitary nodule (1.5 cm)	/	Stable	Liver resection
[9]^c.130dup,pLeu44fs^	F^no detection^		13	Insulin	7.7	16	/	None, MRI discovery	Multiple nodules (5–8 cm)	/	/	/

*F* Female, *BMI* body mass index, *GD* gestational diabetes, *M* male, *y* years old, *US* ultrasonography, / not available

Superscript in the first column of the table represents germline mutations; Superscript in the second column of the table represents somatic mutations

### Biallelic inactivation of *HNF1A*

**Fig. 5:**
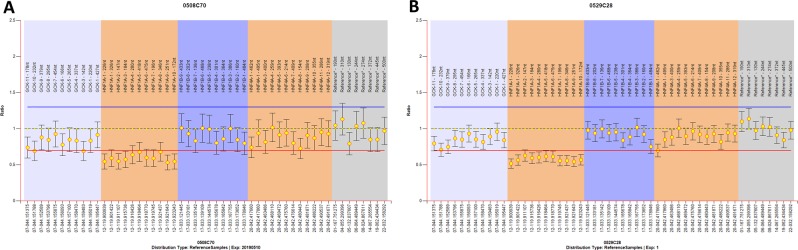
Results of the MS-MLPA in the region of *HNF1A* in proband 1 (**a**) and proband 2 (**b**). The methylation-specific multiplex-ligation-dependent probe amplification (MS-MLPA) assay was performed with SALSA MLPA Probemix P241-E1 MODY Mix 1 (MRC-Holland, Amsterdam, The Netherlands). The P241-E1 MODY Mix 1 Probemix contains 52 MLPA probes with amplification products between 130 and 500 nt. It contains probes for the *HNF4A, GCK, HNF1A*, and *HNF1B* genes and is therefore specific for MODY 1, 2, 3, and 5. For the *HNF4A* gene, 12 probes are included, furthermore 11 for the *GCK* gene, 11 for the *HNF1A* gene, and 10 for the *HNF1B* gene are included. In addition, eight reference probes are included in this probe mix. The identity of the genes detected by the reference probes is available online (www.mlpa.com). The validation experiment should result in a standard deviation <0.10 for all probes over the experiment (0.08 for proband 1, 0.05 for proband 2). The threshold for chromosomal abnormalities was established as follows: the lower limit was 0.7 for deletion and the upper limit was 1.33 for duplication. Abnormal values were plotted outside the threshold line. Heterozygous deletion mutation in exons 1–10 of *HNF1A* gene was found in both proband 1 and proband 2

The two probands were investigated for somatic alterations of *HNF1A* in their liver-cell tumors. Sanger sequencing found no abnormalities; thus, MS-MLPA was performed to search for the variants in *HNF1A*. A heterozygous deletion mutation in exons 1–10 of the *HNF1A* gene was found in both patients (Fig. 5).

## Discussion

We herein describe the occurrence of HCA in MODY 3 patients from two Chinese families. *HNF1A* gene alterations were identified as heterozygous germline mutations in peripheral blood cells and as biallelic inactivated variants in liver adenoma cells in two affected patients. To further evaluate the connection of HCA and MODY 3, we reviewed the relevant literature published in English to date [6–9].

HNF1A is a transcription factor that is expressed in a series of tissues, including the pancreas, liver, and digestive tract. However, the role of *HNF1A* in liver function and proliferation has not yet been elucidated. Studies in mice have reported that *HNF1A* inactivation can result in liver enlargement and hepatic dysfunction [13], and the expression of *HNF1A* decreased in preneoplastic nodules[14]; thus, *HNF1A* was believed to play an important role in cellular growth in liver nodules. In addition, microRNA-122 (miR-122) is the most abundant miRNA in the liver, and its deregulation is associated with hepatocellular carcinoma metastasis. Interestingly, miR-122 is under the transcriptional control of *HNF1A*. These results further indicate the strong correlation between *HNF1A* and liver adenomas.

*HNF1A* mutations are found in ~30% of HCAs; however, most of these patients do not suffer from MODY 3. The mutations are biallelic in most cases of HCA, which is consistent with the role of *HNF1A* as a tumor suppressor [5]. Exons 2–3 of *HNF1A* contain the majority of the DNA binding domain, and mutations in these positions may result in a dominant-negative effect on *HNF1A* function, thus leading to the high penetrance of HCA in carriers of those specific mutations. In the current study, we found biallelic mutations in the DNA binding domain of *HNF1A* in the tumor tissues of the two probands. However, this phenomenon was only observed in two patients suffering from both HCA and MODY 3 in the literature [8]. Further research on the molecular mechanism may be expected to explain this appearance. In addition, germline missense mutations in *HNF1A* may occur outside the dimerization domain in patients with MODY 3; therefore, the encoded proteins may dimerize with the wild-type protein but disrupt transcriptional function [15]. Consistent with the results, the two germline mutations found in our study were located beyond the dimerization domain, and have been reported as disease-causing variants in former studies [16, 17]. The missense mutation c.686G>A changes the residues in the homeodomain region of the protein that are highly conserved in human, rat, mouse, hamster, chicken and salmon [16]. The splicing site mutation c.526+1G>A occurrs within the DNA-binding domain, and is conserved across the rat, mouse, hamster, chicken, xenopus, and salmon [17]. These findings raise the possibility that a particular loss of function mutational spectrum in *HNF1A* may be associated with the development of HCA.

To date, there are a total of four case reports describing ten patients with MODY 3 and HCA [6–9]; the exact prevalence of HCA in MODY 3 patients is still unknown. Our team consecutively recruited MODY patients in the outpatient clinic of Endocrinology at Peking Union Medical College Hospital, Beijing, China; between January 2014 and December 2018 [18], there were a total of 25 patients from 11 families identified as having MODY 3, among which only two patients were identified with HCA (8%), and this result reflects the incomplete penetrance of liver tumors in MODY 3 individuals. Despite a large tumor burden, our patients did not have abdominal symptoms or complaints, and there were no significant liver function abnormalities. These results highlighted the importance of abdominal ultrasound for MODY 3 patients. Furthermore, by reviewing the features of the 12 patients from our study and previous case reports, we found that the clinical expression of HCA was highly variable, from silent to hemorrhage, and the course of HCA was either stable or associated with complications that required surgery. In addition, the patients demonstrated a wide range of ages at diagnosis, from adolescence to late onset after age 50. Notably, most of the patients received well-controlled glucose with HbA1c 5.7–7.7%; thus, there is no evidence that the liver phenotype worsened the severity of diabetes. In contrast with solitary liver adenoma, where nearly 90% of female patients have a history of using contraceptives [3], there was no apparent environmental factor found in our patients and in the publicly reported cases retrieved from the literature with phenotypes of MODY 3 and HCA.

In summary, our study enabled us to evaluate the cosegregation of HCA and MODY 3 with loss of function mutations of *HNF1A*, and advised systematic screening of HCA through noninvasive liver imaging in MODY 3 pedigrees. In the future, additional studies may help to establish the prevalence of HCA in MODY 3 patients and the potential mechanism beneath this connection.

## Supplementary information

Supplementary Table 1
