# Supplementary material for: Primary hepatocellular adenoma due to biallelic HNF1A mutations and its co-occurrence with MODY 3: case-report and review of the literature
Source: Endocrine. 2019 Nov 21;67(3):544–51. doi: 10.1007/s12020-019-02138-x (PMC7054351; doi:10.1007/s12020-019-02138-x)
Supplement: Supplementary file 1 — Supplementary Table 1 [file 12020_2019_2138_MOESM1_ESM.doc]

| **Supplementary Table 1. Probes for *HNF1A*** | | | | | |
| --- | --- | --- | --- | --- | --- |
| **Length (nt)** | **SALSA MLPA probe** | ***HFN1A* exon** | **Ligation site^(a)^ NM_000545.6** | **Partial sequence^(b)^ (24nt adjacent to ligation site)** | **Distance to next probe** |
|  |  | Start codon | 202-204 (ex 1) |  |  |
| 226 | 07708-L07440 | Exon 1 | 210-211 | GCCATGGTTTCT-AAACTGAGCCAG | 0.5kb |
| 326 | 16752-L20209 | Exon 1 | 169 nt after exon 1 | CTTGGAGGTTTG-AGCCTCCAGCCC | 9.7kb |
| 147 | 07710-L07442 | Exon 2 | 639-640 | CACCTGTCCCAA-CACCTCAACAAG | 4.6kb |
| 184 | 07711-L07443 | Exon 3 | 782-783 | AGGTGATGAGCT-ACCAACCAAGAA | 0.7kb |
| 280 | 16907-L21371 | Exon 4 | 1023-1024 | CGGCGCAAAGAA-GAAGCCTTCCGG | 2.0kb |
| 418 | 07713-L07445 | Exon 5 | 1197-1198 | GAGACTGCAGAA-GTACCCTCAAGC | 0.3kb |
| 475 | 16913-L29803 | Exon 6 | 1374-1375 | AGCTTGGAGCAG-ACATCCCCAGGC | 1.0kb |
| 196 | 07715-L07447 | Exon 7 | 1631-1630 reverse | GCACAGGTGGCA-TGAGCGGCTGCT | 1.7kb |
| 346 | 09856-L30357 | Exon 8 | 1717-1716 reverse | CACCTCGGGCTT-GTGGCTGTAGAG | 0.2kb |
| 251 | 07717-L07449 | Exon 9 | 1836-1835 reverse | GCCTCAGTGTCT-GAGGTGAAGACC | 1.6kb |
| 172 | 07718-L07450 | Exon 10 | 1985-1986 | CTCCAGCAGCCT-GGTGCTGTACCA |  |
|  |  | Stop codon | 2095-2097(ex 10) |  |  |

(a) Ligation sites of the P241 MODY Mix 1 MLPA probes are indicated according to RefSeq sequence NM_000545.6, containing 10 exons.

(b) Only partial probe sequences are shown. Complete probe sequences are available at www.mlpa.com.
